# Improved Fetal Magnetic Resonance Imaging Using a Flexible Metasurface

**DOI:** 10.1002/nbm.70016

**Published:** 2025-02-25

**Authors:** Vladislav Koloskov, Viktor Puchnin, Evgeniy Koreshin, Anna Kalugina, Wyger Brink, Polina Kozlova, Irina Mashchenko, Alena Shchelokova

**Affiliations:** ^1^ School of Physics and Engineering ITMO University St. Petersburg Russian Federation; ^2^ Magnetic Detection & Imaging Group, TechMed Centre University of Twente Enschede the Netherlands; ^3^ Department of Radiology Almazov National Medical Research Center St. Petersburg Russian Federation

**Keywords:** dielectric artifacts, fetal imaging, fetus brain, magnetic resonance imaging, metasurfaces, passive shimming

## Abstract

Recent advancements in magnetic resonance imaging (MRI) techniques are promising for the detection of fetal abnormalities, and MRI may supplement or replace prenatal ultrasound scans in the future. In particular, the interest of scientific and medical communities in high‐field (3T) MRI continues to grow due to its improved contrast‐to‐noise and signal‐to‐noise ratios compared to clinical MRI of lower field strength (1.5T). However, 3T MRI shows more prominent dielectric artifacts due to constructive and destructive interference of standing waves inside the body at these frequencies. Here, we present a concept of passive radiofrequency shimming using metasurface‐based pads to improve image quality in fetal MRI at 3T. The proposed metasurface increases the efficiency and homogeneity of the radiofrequency magnetic field, reducing dielectric artifacts in the fetal body and brain images. We offer an ultralight and compact passive way to improve 3T imaging of fetal brain and body structures, simplifying clinical workflows and decreasing the procedure time.

## Graphical abstract

A flexible metasurface pad, positioned between the MRI body coil and the patient, enhances the efficiency and uniformity of the radiofrequency magnetic field. As a result, it reduces fetal and brain dielectric artifacts, which typically appear as signal voids in the images. This innovative approach enables lightweight and compact imaging of fetal brain and body structures at 3T, while also shortening the procedure time.

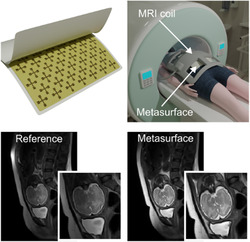

**Abbreviations**
BMI: body mass index
CDH: congenital diaphragmatic hernia
Cv: coefficient of variation
FOV: field of view
FSE: fast spin echo
GW: gestational week
MIP: maximum intensity projection
PAS: placenta accreta spectrum
PCB: printed circuit board
ROI: region of interest
pTx: parallel transmission
SAR: specific absorption rate
SAR_wb_
: whole‐body specific absorption rate
SAR_10g_
: maximum specific absorption rate averaged over 10 g of tissue
SNR: signal‐to‐noise ratio

## 1 Introduction

Noninvasive fetal diagnostic techniques are essential in the management and monitoring of pregnancy and the detection of fetal abnormalities. Until the 1990s, prenatal ultrasound was the only clinically acceptable method of fetal imaging. However, in the past two decades, fetal magnetic resonance imaging (MRI) has become feasible due to advances in magnetic resonance (MR) technology [1]. As a result, the use of fetal MRI as a supplementary tool for evaluating fetal anatomy in clinical practice increased [2]. MRI provides high‐contrast images in three dimensions, comprehensively visualizing the anatomy and function of the fetus and extrafetal structures and correctly diagnosing pregnancies with congenital anomalies [3]. In addition, fetal MRI allows investigating various aspects of amniotic fluid and diagnosing disorders of both the mother and fetus [4]. In other words, MRI can provide high‐quality diagnostic information in addition to prenatal ultrasound [2].

MRI with a static magnetic field strength of 1.5T and 3T has become the most widely used technique for fetal imaging [5, 6]. In particular, 3T MRI is of increasing interest due to its higher signal‐to‐noise ratio (SNR), which leads to more accurate visualization of anatomical structures and abnormalities [7]. However, a fundamental limitation of the higher magnetic field strength is the pronounced dielectric artifact caused by the standing wave phenomenon, resulting in nonuniform contrast in the images [6]. At 3T, the radiofrequency (RF) wavelength inside the body becomes comparable to the size of the abdominal area, leading to constructive and destructive interference areas in the RF transmit field (B_1_^+^). This effect is particularly problematic during pregnancy due to the larger size of the abdomen and the high conductivity and permittivity of the amniotic fluid surrounding the fetus [8]. The artifacts also become more pronounced in the third trimester of pregnancy and for multiple pregnancies [7].

There are several techniques compensating for these artifacts, such as active and passive shimming of the B_1_^+^ field [9, 10, 11, 12]. The most advanced method of RF magnetic fields tailoring is parallel transmission (pTx), where RF shimming is the most common option. RF shimming enables adjusting the amplitude and phase of each channel of a multichannel transmit RF coil to improve the B_1_^+^ field distribution in the area of interest [13, 14]. Several MRI vendors offer 3T MRI scanners with dual‐channel pTx systems, which are available for human imaging [15]. Nevertheless, MR scanners without active shimming are still used in many clinical institutions worldwide, and a different approach is required to address these artifacts.

Passive shimming approaches have been introduced in various applications of high‐field MRI, efficiently correcting B_1_^+^ field inhomogeneities [16]. In most works, a high‐permittivity material is placed near the region of interest (ROI), and the desired RF magnetic field is obtained with a secondary RF magnetic field produced by the displacement currents induced within the material. These currents are generated by the incident electromagnetic field excited by the RF transmit coil, hence the term “passive” shimming. State‐of‐the‐art high‐permittivity dielectric pads have been utilized for passive shimming of the RF magnetic field for different anatomical areas: extremities [17], brain [18], abdomen [19], and fetal imaging [20]. When developing such pads, their geometrical and electromagnetic properties should be adjusted to obtain the desired effect [12, 21]. This can result in a heavy and bulky structure in applications with a large field of view (FOV), such as abdominal imaging [19]. This can be problematic in fetal imaging, where the patient's comfort is often already compromised. Another limitation of dielectric materials is the degradation of their physical and electrical parameters over time.

As an alternative to high‐permittivity dielectric pads, artificial metal‐dielectric structures (metamaterials and metasurfaces) have been proposed [22, 23, 24, 25]. For instance, to visualize the abdominal area, dielectric pads can be replaced by a compact and flexible metasurface to alter the distribution of B_1_^+^ and consequently improve the image quality [26]. The proposed metasurface consisted of a two‐dimensional periodic structure of metallic flat crosses connected by parallel‐plate capacitors printed on an ultrathin dielectric substrate. The main advantages of such devices include compactness, lightweight, and durability. Furthermore, the properties of metasurfaces are defined by their geometry and can be optimized depending on the optimization goal or region of interest [27].

In this work, we present and characterize a metasurface‐based passive shimming approach to improve fetal imaging at 3T. The metasurface design is based on a two‐dimensional grid of cross‐shaped cells connected via capacitors. The design is optimized for the fetus using electromagnetic simulations and implemented using an ultralight and flexible printed circuit board (PCB). The effect of the metasurface on the RF magnetic field, specific absorption rate (SAR), and temperature is evaluated numerically using body models at the 28th and 36th gestational weeks (28GW and 36GW). In addition, we evaluate the metasurface's effect on in vivo imaging in seven patients and demonstrate the technique's potential for fetal imaging at 3T.

## 2 Methods

### 2.1 Metasurface Design

**FIGURE 1:**
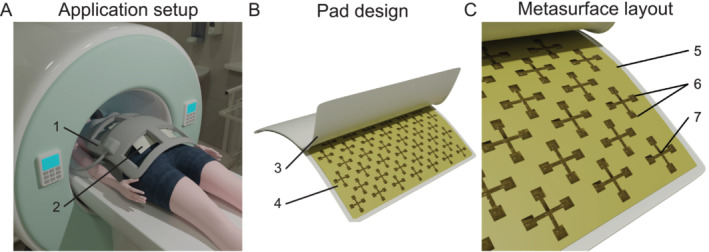
(A) Conceptual image of MRI scanning of the abdominal cavity with passive shimming: The receive body array (1) is placed on a volunteer with the pad (2) on the fetal area. (B) A conceptual image of a case (3) with metasurface (4). (C) Metasurface design (top layer): dielectric substrate (5) with parallel plate capacitors (6) connected by metal crosses (7).

The MRI studies with pregnant women using a metasurface are illustrated in Figure 1A. During fetal MRI, the patient usually lies in a supine position or in some cases on the side. The integrated body birdcage coil is used for signal transmission, and a body receive array is used for signal reception. The receive coil (1, Figure 1A) and the metasurface (2, Figure 1A) positioned underneath the coil are centered on the fetus and placed above the investigated region.

This work uses a previously proposed metasurface design for passive B_1_^+^ field shimming in the abdomen region [26]. The metasurface includes two metal grids of cross‐shaped cells connected via parallel plate capacitors, one on each side of the dielectric substrate (lumped capacitors could also be employed). Typically, one would design a metasurface or passive RF shim to cover at least the ROI to match the area where the field needs to be tailored. Consequently, the overall dimensions of our proposed design are based on the average fetus size. The metasurface design used in our work is presented in Figure 1B,C. When the metasurface is placed inside the transmit RF coil, the incident field excites conduction currents in the metal grid, which modify the field distribution near the metasurface. In Figure S1, the magnetic field distribution of the first resonant mode of the metasurface is shown in three orthogonal planes.

Because the metasurface is a periodic structure, we can adjust the number of unit cells and capacitance to achieve the desired field correction within the ROI. Here, we aimed to eliminate the B_1_^+^ field minima in the fetus by enhancing the RF magnetic field in this area. As the optimization function, we chose the coefficient of variation (Cv) of the root‐mean‐squared B_1_^+^ field in the ROI. Cv was calculated as the ratio of the standard deviation to the mean value of the B_1_^+^ field amplitude in the desired ROI. As a starting point of the optimization process, the overall dimensions of the structure were set to 300 × 300 mm^2^ according to the ROI. Then, we determined the cell size and capacitance within each cell. The number of unit cells varied from 2 to 15; consequently, the cell size changed from 150 × 150 mm^2^ to 20 × 20 mm^2^. We should note that cells smaller than 20 × 20 mm^2^ might increase the coupling between the patch capacitors and copper stripes [23, 24]. The capacitance was swept from 5 to 55 pF with a 5‐pF step. The metasurface optimization is described in detail in Metasurface Optimization in the Supporting Information.

### 2.2 Electromagnetic Simulation

We performed electromagnetic analyses using CST Studio Suite 2021 (Dassault Systèmes Simulia Corp., Vélizy‐Villacoublay, France). A generic RF transmit coil was modeled as a 16‐leg high‐pass birdcage coil with a length of 650 mm and a diameter of 700 mm. The model was tuned and matched to 50 Ohms at 123 MHz and excited in quadrature. We used two voxelized body models of pregnant women at 28GW and 36GW with a voxel size of 5 × 5 × 5 mm^3^ (Sim4Life Virtual Population, Switzerland). For each voxel model, we investigated three setups: (1) a reference case, (2) with a state‐of‐the‐art dielectric pad [12], and (3) with the metasurface. For both gestation periods, both the dielectric pad and the metasurface were centered on the fetus. The metasurfaces' initial geometrical dimensions and electromagnetic properties were based on the reference dielectric pads that were optimal for minimizing the Cv in the fetus's body [12]. A dielectric pad with dimensions of 292 × 299 × 15 mm^3^ and *ε*_*r*_ = 270 was used for the voxel model at the 28GW. A dielectric pad with dimensions of 247 × 292 × 15 mm^3^ and *ε*_*r*_ = 248 was used for the voxel model at the 36GW. For dielectric pads, the material's conductivity was set to 0.2 S/m. Once the parameters of the metasurfaces were optimized (see Metasurface Optimization in the Supporting Information), we evaluated their effect on the whole‐body SAR (*SAR*_*wb*_); local SAR, defined as the maximum SAR averaged over 10 g of tissue (*SAR*_10*g*_); and SAR efficiency, evaluated as the ratio of mean B_1_^+^ field in the ROI to the square root of the maximum *SAR*_10*g*_. Moreover, we simulated the temperature distribution in the body using a thermal solver in CST Studio Suite 2021 to evaluate the metasurface's impact on heating. The tissue temperature was modeled for a total exposure duration of 300 s (the average duration of one MR scan) [28, 29], with an initial tissue temperature of 36.6°C and a blood temperature of 37°C. According to international standards [30], the spatially localized temperature limit is 39°C in the torso for the normal operating mode (*SAR*_*wb*_ < 2 W/kg), so the temperature rise should not exceed 2.4°C with respect to 36.6°C [31]. The input power was normalized to obtain a *SAR*_*wb*_ of 2 W/kg. We evaluated the maximum temperature change in the fetus, placenta, and amniotic fluid regions with the simulated temperature distribution. In other words, we estimated the upper temperature limit that might be induced in the ROIs with a dielectric pad or metasurface. All the numerical simulation results were postprocessed in MATLAB (Version 2022b; The Mathworks, Inc, Natick, MA).

### 2.3 Metasurface Manufacturing

The optimized metasurface was manufactured as two metallization layers separated by a flexible dielectric substrate with a relative permittivity of *ε*_*r*_ = 3.4 (DuPont™ Pyralux® AP8515R; DuPont de Nemours, Inc., Wilmington, DE). To make the structure flexible and compact, we implemented discrete capacitors as parallel‐plate capacitors according to the formula *C* = *ε*_0_ *ε*_*r*_ *S*/*d*, where *ε*_*r*_ and *d* are the relative permittivity and thickness of the substrate, respectively. The total thickness of the metasurface was 61 μm (the dielectric substrate was 25 μm thick, and two metallization layers were 18 μm thick each). The relative permittivity *ε*_*r*_ could vary up to 10% from the nominal value, resulting in the capacitance variation. We placed the structure inside a leather case to protect the metasurface from physical damage and avoid direct contact with the patient's skin (3, Figure 1B).

### 2.4 In Vivo Study Design

Experimental studies were performed on a 3T MRI system (MAGNETOM TrioTim; Siemens Healthineers, Erlangen, Germany) at Almazov National Medical Research Center (St. Petersburg, Russian Federation) using a quadrature transmit integrated birdcage coil and a six‐channel body coil for signal reception. The study was approved by the Almazov National Medical Research Center's local ethics committee (Protocol Number 1710‐21), and written informed consent was obtained from all the subjects before the experiments. The study population consisted of seven pregnant female patients with mean body mass index (BMI) of 25.7 ± 1.7 (minimum, 23.6; maximum, 27.9) kg/m^2^, age of 34 ± 5 (minimum, 26; maximum, 40) years, and gestation period of 33 ± 4 (minimum, 27; maximum, 39) weeks. The details of the studied patients are presented in Table 1. MRI of these subjects was performed according to the following clinical indications [32]:

1. If the use of ultrasound is difficult (due to maternal obesity, scars, lack of water, and challenges in visualization in the second or third trimester), MRI can be performed at any time when diagnostic images are required.
2. If malformations of the central nervous system and/or internal organs of the fetus are suspected (e.g., congenital diaphragmatic hernia, CDH) according to ultrasound findings or in case of intrauterine death of one of the fetuses in multiple pregnancies, MRI should be performed not earlier than at the 18GW.
3. In the case of extrafetal abnormalities (e.g., placenta accreta spectrum disorders, PAS) or the need for MR pelvimetry before the delivery, MRI should be performed not earlier than the 24GW.

**TABLE 1:** Summary of the characteristics of the patients that participated in the study.

Patient ID	Patient's age	Gestational week	Clinical indication
1	26	29	PAS2‐3
2	37	39	PAS2‐3
3	34	35	PAS2‐3
4	29	37	CDH
5	36	32	PAS2‐3
6	39	27	PAS2‐3
7	40	37	MR pelvimetry
8	38	32	PAS2‐3

Abbreviations: CDH, congenital diaphragmatic hernia; PAS, placenta accreta spectrum.

Fetal MRI was performed using a multislice single‐shot *T*_2_‐weighted fast spin echo (FSE) sequence acquired in all three anatomical planes. The following parameters were used for images in the coronal plane: *TR*/*TE* = 2500/95 ms, FOV = 350 × 350 mm^2^, refocusing tip angle = 150°, echo train length = 256, matrix = 320 × 256, slice thickness = 5 mm, slice gap = 0 mm, and number of slices = 31. For images in the transverse and sagittal planes, the parameters were the following: *TR*/*TE* = 1500/94 ms, FOV = 380 × 280 mm^2^, refocusing tip angle = 150°, echo train length = 256, matrix = 320 × 170, slice thickness = 5 mm, slice gap = 0 mm, and number of slices = 35. The metasurface and the receive coil were centered on the fetus (as shown in Figure 1A). The MRI complied with all the safety rules and consisted of two consecutive stages. First, in the experiment with the metasurface, the sequence was acquired in three orthogonal planes. The metasurface was then removed, and the protocol was repeated. SNR maps were obtained by dividing *T*_2_‐weighted anatomical images by the standard deviation of noise measured in the background and multiplied by the 0.66 Rayleigh distribution correction factor [33]. Finally, we performed an experimental study in one additional patient at a gestation period of 32 weeks using a 3T MRI system that supports dual‐channel active B_1_^+^ shimming (MAGNETOM Vida; Siemens Healthineers, Erlangen, Germany). Here, we used a multislice single‐shot *T*_2_‐weighted FSE sequence acquired in all three anatomical planes. The following parameters were used for images in the coronal and sagittal planes: *TR*/*TE* = 1000/78 ms, FOV = 380 × 380 mm^2^, refocusing tip angle = 128°, echo train length = 111, matrix = 320 × 259, slice thickness = 4 mm, slice gap = 0.8 mm, and number of slices = 41 and 35. For the transverse images, the following pulse sequence parameters were set: *TR*/*TE* = 1050/78 ms, FOV = 380 × 380 mm^2^, refocusing tip angle = 90°, echo train length = 111, matrix = 320 × 259, slice thickness = 4 mm, slice gap = 0.8 mm, and number of slices = 51. Dual‐channel B_1_^+^ shimming was enabled with a shim ROI centered at the fetus. In this case, we did not use the metasurface or dielectric pad. We compared the results for this subject with those for a subject with the same gestation period (32 weeks) obtained with the TrioTim system with and without the metasurface.

### 2.5 Qualitative Image Analysis

The *T*_2_‐weighted images with and without the metasurface were evaluated and compared by three independent radiologists with work experience of 5 years (Expert 1), 5 to 10 years (Expert 2), and more than 10 years (Expert 3). The first group of MR data consisted of the results acquired without the metasurface and the second group of the results acquired with the metasurface pad. Then, the two groups were randomized and presented for evaluation. The image quality was assessed using a 5‐point Likert scale: 1, *insufficient quality*; 2, *poor quality*; 3, *medium quality*; 4, *good quality*; and 5, *excellent quality*. We asked the radiologists to do the following:

- Please evaluate the general diagnostic quality of the images: Can these images be used in clinical practice?
- Please evaluate the quality of the images in terms of absence/presence of a dielectric artifact.
- Please evaluate the images regarding the visualization quality of the extrafetal and fetal structures.

## 3 Results

### 3.1 Electromagnetic Simulation

We found the optimal set of metasurface parameters that can be used for both gestation periods. The optimized structure had dimensions of 300 × 300 mm^2^, 15 × 15 unit cells, a unit cell size of 20 × 20 mm^2^, and 30 pF capacitance; these parameters were used for further qualitative and quantitative numerical analysis.

**FIGURE 2:**
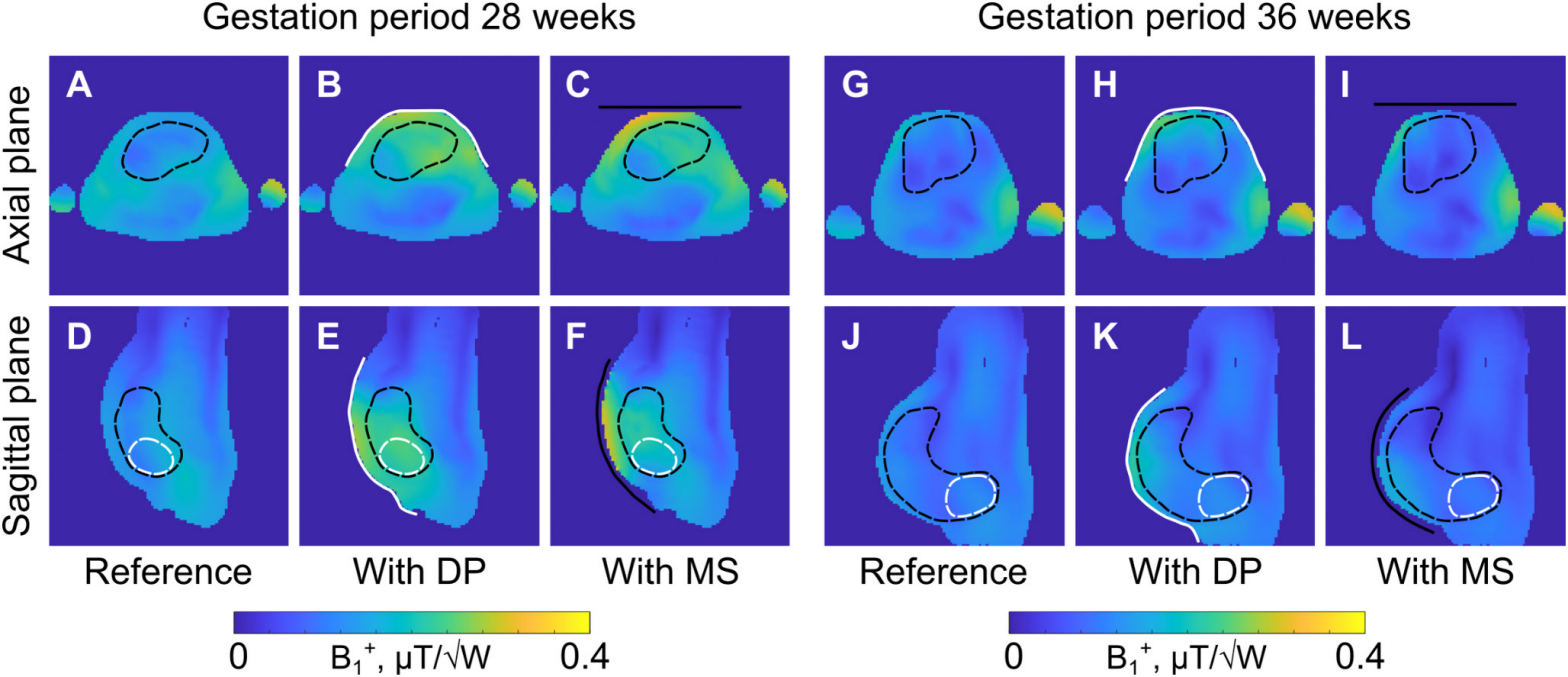
Numerically calculated B_1_^+^ field maps for the female voxel models at the 28GW (7th month of gestation) for the reference setup (A,D), with the dielectric pad (B,E), and with the metasurface (C,F), and at the 36GW (9th month of gestation) for the reference setup (G,J), with the dielectric pad (H,K), and with the metasurface (I,L). The simulated fields are normalized to 1 W of accepted power. Black and white dashed lines denote the fetus's body and brain, respectively. White and black lines show the position of the dielectric pad and metasurface, respectively. DP ‐ dielectric pad, MS ‐ metasurface.

Figure 2 shows simulated B_1_^+^ maps for the voxel model at the 28GW and 36GW for three cases: (1) birdcage coil only (reference case), (2) with the conventional dielectric pad, and (3) with the metasurface placed on the top of the female model abdomen near the fetus. The dielectric pad and the proposed metasurface similarly improved the B_1_^+^ field amplitude in the ROI for the body model at the 28GW compared to the reference case (Figure 2A,D). These changes can be observed within the black dashed region denoting the fetus's body in the axial and sagittal planes in Figure 2B,C and Figure 2E,F, respectively. One can also note in Figure 2F that with the metasurface, the B_1_^+^ field amplitude is slightly lower around the distal edge of the metasurface compared to that with the dielectric pad. If this happens near the fetus's brain, the metasurface can be repositioned. In the 36th GW body model, the effects of the dielectric pad (Figure 2H,K) and metasurface (Figure 2I,L) on the B_1_^+^ are less pronounced within the fetus region compared to the reference case (Figure 2G,J) but remain rather localized near the metasurface.

Quantitative assessments of the B_1_^+^ field for both voxel models are shown in Table 2. At 28GW, the dielectric pad and metasurface improved the B_1_^+^ field amplitude in the fetal body by 38% and 28%, respectively. Moreover, compared to the reference case, metasurface improved the B_1_^+^ homogeneity (i.e., 1 − Cv) by 2.1%pt in the fetal body. In contrast, no improvement was observed in the case of the dielectric pad. In the fetal brain, the dielectric pad improved the B_1_^+^ field homogeneity by 9.7%pt whereas the metasurface only by 2.3%pt.

**TABLE 2:** Numerically calculated results of transmit and SAR characteristics for the fetus's body and brain for 1 W of total accepted power.

	Fetus body			Fetus's brain	
	B_1_^+^, uT	Cv, %	SAR efficiency, uT/√W/kg	B_1_^+^, uT	Cv, %
**28th gestational week**					
Reference	0.133	20.6	0.347	0.147	17.9
Dielectric pad	0.183	22.5	0.470	0.230	8.2
Metasurface	0.170	18.5	0.410	0.174	15.6
**36th gestational week**					
Reference	0.102	19.4	0.233	0.111	9.6
Dielectric pad	0.125	25.9	0.284	0.118	5.8
Metasurface	0.104	31.5	0.213	0.095	8.3

At 36GW, the dielectric pad increased the B_1_^+^ field amplitude by 23% and the B_1_^+^ homogeneity by 6.5%pt, whereas for the metasurface, the B_1_^+^ field amplitude remained the same, and the B_1_^+^ inhomogeneity increased by 12.1%pt. In the fetal brain, the mean B_1_^+^ increased from 0.111 uT in the reference case to 0.118 uT for the dielectric pad and decreased to 0.095 uT for the metasurface. At the same time, the B_1_^+^ field homogeneity was improved by 3.8%pt and 1.3%pt, respectively.

**FIGURE 3:**
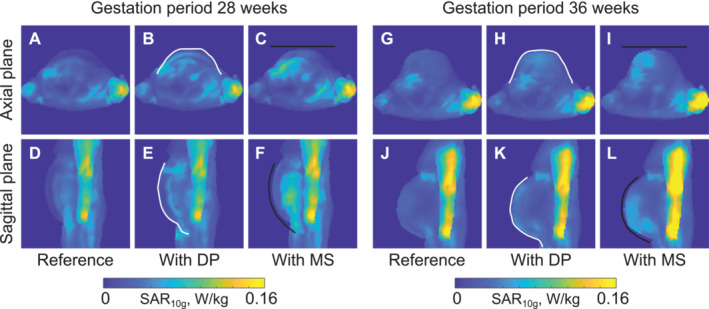
Numerically calculated MIP SAR_10g_ maps for female voxel models at the 28GW (7th month of gestation) for the reference setup (A,D), with the dielectric pad (B,E), and with the metasurface (C,F), and at the 36GW (9th month of gestation) for the reference setup (G,J), with the dielectric pad (H,K), and with the metasurface (I,L) for 1 W of accepted power. White and black lines show the position of the dielectric pad and metasurface, respectively. DP ‐ dielectric pad, MS ‐ metasurface.

Figure 3 shows maximum intensity projections (MIP) of the 10‐g‐averaged SAR distribution for the voxel model at 28GW and 36GW. In the axial plane, there are hotspots in the right arm for all the investigated setups, with a slight increase for the metasurface. As for other areas, such as the fetus, placenta, and amniotic fluid, deviations in SAR values were observed, not exceeding 30% for the metasurface compared to the reference case (see details in Table S1).

SAR efficiency results for both voxel models are shown in Table 2. The dielectric pad and metasurface improved SAR efficiency by 35% and 18% for the 28‐week‐old fetus, and for the 36‐week gestational period, a 22% increase and 9% decrease were found, respectively. The increase in SAR efficiency for the metasurface is lower than for the dielectric pad. One of the main reasons is the proposed structure has lower losses than the conventional pad. In the case of 36GW, the decrease in SAR efficiency for the metasurface is partly explained by the almost negligible improvement of the B_1_^+^ field due to the nonconforming metasurface model.

**FIGURE 4:**
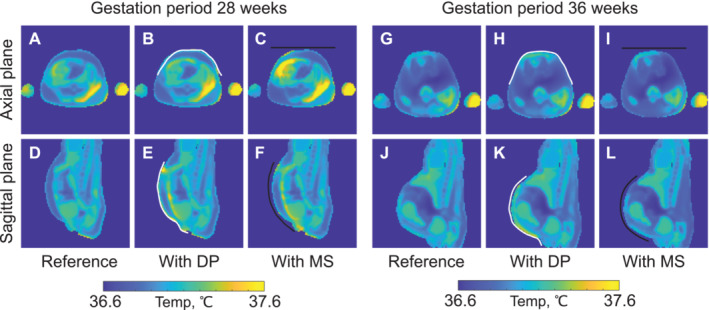
Numerically evaluated temperature maps after 300 s of excitation for female voxel models at the 28GW (7th month of gestation) for the reference setup (A,D), with the dielectric pad (B,E), and with the metasurface (C,F), and at the 36GW (9th month of gestation) for the reference setup (G,J), with the dielectric pad (H,K), and with the metasurface (I,L). The accepted power is specified for each setup to obtain a SAR_wb_ of 2 W/kg. The initial tissue temperature was set to 36.6°C. White and black lines show the position of the dielectric pad and metasurface, respectively. DP ‐ dielectric pad, MS ‐ metasurface.

The results of the thermal simulations are shown in Figure 4 and Table 3. We evaluated the maximum temperature increase in the torso, including fetus, placenta, and amniotic fluid regions, as the maximum difference between temperature for the dielectric pad or metasurface and the reference case when normalized to a *SAR*_*wb*_ of 2 W/kg. Numerical simulations showed that for the voxel model at the 28GW and 36GW, with the dielectric pad, the local temperature increase was found in the skin by up to 1°C and 0.57°C, and with the metasurface, it was found in the amniotic fluid up to 0.8°C and 0.3°C compared to the reference configuration, respectively. It is worth noting that this calculation is an upper estimate. If one calculates the thermal exposure for the mean B_1_^+^ field amplitude in the fetus ROI for all cases, the upper bound of temperature increase for the voxel model at the 28GW can be minimized to 0.4°C and 0.3°C for the dielectric pad and metasurface, respectively, as less input power will be used. Additionally, as the dielectric pad was in contact with the voxel model with no air gap, it resulted in a higher temperature rise in the ROI compared to the setup with the metasurface, with a small air gap between the structure and the body model. On the other hand, when an air gap was introduced between the dielectric pad and the voxel model, the temperature rise was less prominent, but the B_1_^+^ efficiency decreased compared to the case with no air gap (see more details in the Supporting Information).

**TABLE 3:** Thermal simulation results.

Considered setup	Accepted power, W	B_1_^+^, uT	SAR_wb_, W/kg	Maximum temperature increase, °C
**28th gestational week**				
Reference	157.2	1.67	2.00	N/A
Dielectric pad	155.2	2.28		1.00
Metasurface	154.8	2.11		0.80
**36th gestational week**				
Reference	163.5	1.30	2.00	N/A
Dielectric pad	161.0	1.58		0.57
Metasurface	156.2	1.30		0.30

### 3.2 Metasurface Prototype

Due to limitations imposed by the manufacturer, the metasurface had to be scaled down to 280 × 280 mm^2^. Consequently, we reduced the number of unit cells from 15 × 15 to 12 × 12 and increased the capacitance from 30 pF to 37 pF. Due to this size reduction, the useful FOV of the metasurface slightly decreased. However, we optimized the design to preserve the improvement in the B_1_^+^ field amplitude and homogeneity. Moreover, the reflection coefficients of the initial metasurface and the reduced one showed their first resonant frequencies are close, 134 and 132 MHz, respectively, above the operational frequency of 123 MHz, as seen in Figure S3.

### 3.3 In Vivo Studies

**FIGURE 5:**
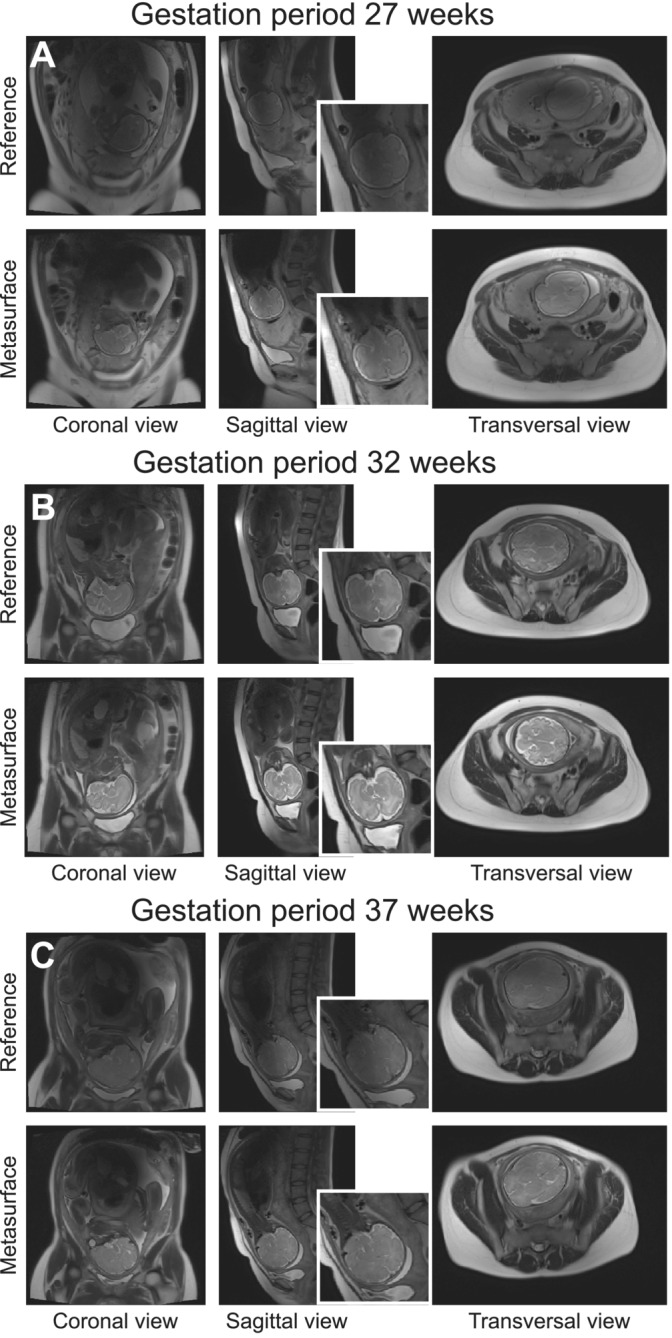
*T*_2_‐weighted images for patients at the 27th (A), 32nd (B), and 37th (C) gestation weeks (7th, 8th, and 9th months of gestation).

Examples of the MR images obtained without and with the metasurface from three patients representing the full range of the investigated gestation periods are shown in Figure 5. The figure includes one subject at the shortest studied gestation period (27 weeks), one subject at the average gestation period (32 weeks), and one subject at the longest gestation period (37 weeks). In the reference case (without active or passive shimming approaches), *T*_2_‐weighted FSE images in three orthogonal planes demonstrate the standing wave effect (i.e., an area of reduced signal that impairs the visualization of fetal and extrafetal structures). With the metasurface, this standing wave artifact was substantially improved. Both fetal and extrafetal structures were better defined (bottom images in Figure 5). Particularly, in the images from patients with placenta previa and PAS disorders, the placenta‐myometrium border could be distinguished better, and the placental invasion was visualized in more detail. In fetal structures, the contours between the gray and white matter of the brain and brain structures filled with cerebrospinal fluid were better defined (insets in Figure 5). *T*_2_‐weighted sagittal images for all patients without and with metasurface can be found in Figure S5 in Image Analysis in the Supporting Information.

SNR maps in Figure S6 show inhomogeneity in the fetal brain region. In the sagittal plane for the 32GW and 37GW, the signal drops due to the standing wave effect. When the metasurface was placed near the fetus, the homogeneity was considerably improved, allowing for visualization of the fine brain structures. SNR in the fetal brain region increased by 50%, 23%, and 10% for the 27GW, 32GW, and 37GW, respectively. Results for all patients are presented in Table S4.

**FIGURE 6:**
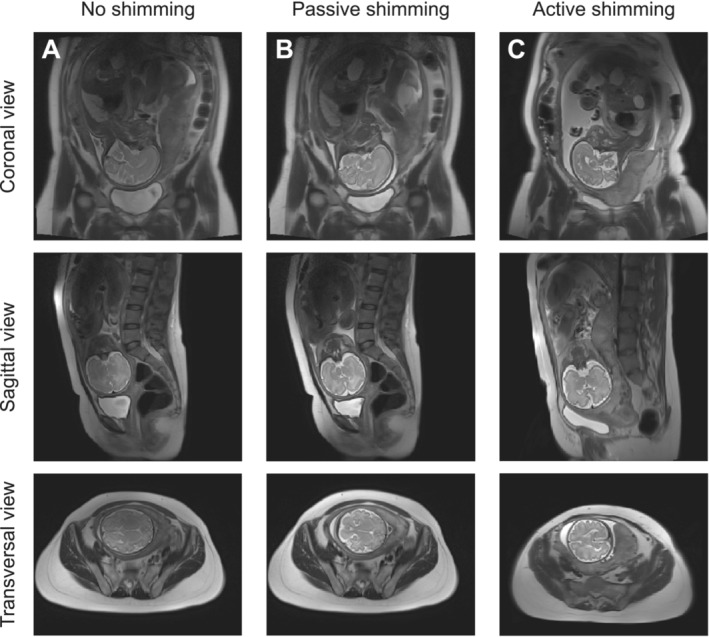
Comparison of *T*_2_‐weighted images for two subjects at the 32nd gestational week. (A) A system driven in quadrature, (B) with a metasurface, and (C) with active B_1_^+^ shimming.

In Figure 6, different shimming approaches are compared. Both passive shimming with the metasurface and active shimming with two‐channel pTx improve the above‐described signal voids. From the images, we estimated signal inhomogeneity in the fetal brain for the reference configuration to be 28%, which was reduced to 19% for both shimming approaches. SNR in the fetal brain region increased from 477 to 587 for the passive shimming approach compared to the case without any shimming, and SNR was found to be 456 for the case with an active shimming approach (Table S4).

### 3.4 Qualitative Image Analysis

The assessment of the diagnostic quality of the obtained MR images is shown in Table 4. Statistics for all the volunteers are presented in detail in Table S5. Based on the opinions of independent experts with different periods of work experience, the MR images of the fetus obtained with a combination of the six‐channel body coil and the metasurface have better diagnostic capabilities compared to standard approach with the body coil only. All fetal and extrafetal structures in the ROIs were well visualized when the metasurface was present. In the other group, the dielectric effect made interpreting the images extremely difficult. It was confirmed by the range of scores for the obtained images: from 3 to 5 points using the six‐channel body coil only and from 4 to 5 points with the metasurface.

**TABLE 4:** Image quality assessment (answers of radiologists to Questions A–C) of the images without and with the metasurface using the Likert scale. Mean (standard deviation) values are presented in the table.

Expert No.	Without metasurface			With metasurface		
	A	B	C	A	B	C
Expert 1	3.63 (0.86)	3.63 (0.48)	3.75 (0.66)	4.50 (0.50)	4.63 (0.48)	4.88 (0.33)
Expert 2	3.25 (0.83)	3.63 (0.70)	3.88 (0.60)	4.38 (0.48)	4.25 (0.43)	4.38 (0.48)
Expert 3	3.50 (0.87)	4.00 (0.50)	3.88 (0.60)	4.25 (0.66)	4.63 (0.48)	4.50 (0.50)
Mean value	3.46 (0.87)	3.75 (0.60)	3.83 (0.62)	4.38 (0.56)	4.50 (0.50)	4.58 (0.49)

*Note:* Letters A–C mark the questions for image assessment: (A) Please evaluate the general diagnostic quality of the images—can these images be used in clinical practice? (B) Please evaluate the quality of the images in terms of the absence/presence of a dielectric artifact. (C) Please evaluate the images regarding the visualization quality of the extrafetal and fetal structures.

## 4 Discussion

Numerical simulations showed that the optimized metasurface design improves B_1_^+^ field distribution within the fetal area (body and brain) for female body models at the 28th and 36th weeks of gestation. Then, the proposed approach was validated in pregnant patients with clinical indications for MR studies. *T*_2_‐weighted images and SNR maps for seven patients at different gestation periods (33 ± 4 weeks) demonstrate that the metasurface is a universal and sustainable tool for improving the image quality for various stages of pregnancy and subject BMIs.

Qualitative analysis of the electromagnetic simulations showed that both passive shimming approaches, that is, dielectric pads and metasurfaces, improved the B_1_^+^ field amplitude and distribution in the fetus ROI (Figures 2 and 3). Although the standing wave artifact was more pronounced in the voxel model at the 36GW compared to the model at the 28GW, both devices improved the B_1_^+^ field amplitude and homogeneity in the fetus, as shown in Table 2. The mean B_1_^+^ field amplitude value in the fetus and the homogeneity in the fetal brain region were improved both by the dielectric pad and metasurface for all the studies patients. However, the dielectric pad performs better in terms of the B_1_^+^ amplitude than the proposed metasurface. This can be associated with the limitations of the modeling and meshing of the structure in the time‐domain solver, which was the case in the previous work devoted to the metasurfaces [34]. Thus, we do not expect the current metasurface to outperform the dielectric pad to improve the amplitude and homogeneity of the B_1_^+^ field distribution inside the ROI. But we anticipate a comparable performance of the optimized metasurface and the dielectric pad regarding SNR, anatomical structure differentiability, and safety.

Notably, the use of the dielectric pad or metasurface affected not only the RF magnetic field but also the associated electric field. Overall, this resulted in a slight increase inside or near the structure with small local increases in SAR when normalized to input power (Figures 2 and 3). This is consistent with earlier reports of dielectric materials [12, 17, 18]. However, the local SAR can be reduced when normalizing to the mean B_1_^+^ field value in the ROI, as less RF power is required to perform the desired flip angle. This was confirmed by estimated SAR efficiency, which increased by least 18% for the metasurface compared to the reference for almost all the cases. Only for the setup with the voxel model at the 36GW with the metasurface, the SAR efficiency dropped by 9%. The key reasons for this deterioration are the localized electric field and low losses of the metasurface compared to the dielectric pads. Moreover, numerical simulations of both structures showed a tiny effect on B_1_^+^ field distribution with the voxel model at the 36GW. In addition, we note that the dielectric pads were form fitting with both voxel models in all directions, whereas the metasurfaces were curved only in the *z* direction. This may reduce the impact on the B_1_^+^ field distribution close to the edges of the metasurface compared to the dielectric pad and an increased inhomogeneity due to a more localized enhancement of the B_1_^+^ field. In that case, the metasurface should be moved closer to the fetal brain. Moreover, the metasurface design was optimized for the whole fetal body as a more general case. Despite this, in the experimental anatomical images obtained with the metasurface, the field distribution is significantly improved for all the investigated gestational periods (from the 26GW to 40GW).

Thermal simulations showed no significant changes in tissue temperature for the dielectric pad or metasurface compared to the reference case (Figure 4 and Table 3). The highest temperature increase for the voxel model at the 28GW was 1°C, whereas for the 36GW, this was 0.8°C [31]. Higher temperature increases were observed when the dielectric pad was used compared to the metasurface, which we attribute to the limitations of the numerical model. The modeled dielectric pad directly interacted with the voxel model. Thus, the produced heat could be partially transferred between the structure and the body. As for the metasurface, the model was not as tight fitting, resulting in a small air gap. Other studies have shown that an air gap may be needed to account for the insulation material usually enclosing a dielectric pad, which may also be relevant for the safety assessment of the dielectric configuration [35]. Despite including SAR assessment and temperature simulations, the current research lacks experimental validation through RF heating measurements [36]. This limitation impedes the immediate application of metasurface in clinical studies. To enable clinical use, FDA approval should be obtained. For that, we need to comprehensively analyze the SAR and temperature distribution for models with varying BMI in the scanner and across a broad range of gestational weeks.

The results obtained in pregnant patients confirmed the numerical predictions with *T*_2_‐weighted anatomical images, showing an enhanced image quality, as confirmed by radiologists. Thus, the metasurface partially or entirely removed the dark regions observed in the transverse plane for patients at the 27th and 32nd weeks of gestation. In the coronal and sagittal planes, there is a higher contrast in the fetus's brain region for all the examined patients. Improved tissue contrast and contrast uniformity are significant when examining fetal brain structures, as they affect the neonatal prognosis. Furthermore, 3T fetal MRI is essential for the detailed evaluation of the placenta and the umbilical cord due to its higher spatial resolution compared to lower strength MRI [37]. Moreover, with the use of the metasurface, the overall imaging procedure becomes faster and, therefore, more convenient for radiology technicians and more comfortable for pregnant patients, which may find it difficult to lie still in a supine posture during the scan.

Additional experimental studies showed that the proposed metasurface‐based passive shimming approach provides field redistribution comparable with active shimming (Figure 6). Consequently, when active solutions are unavailable, one can use metasurfaces to achieve higher homogeneity and SNR in fetal imaging without breaching RF safety limits. This was also shown during the experiment when the objective parameter *SAR*_*wb*_ values were below the upper limit ≤ 2 W/kg established for pregnant subjects in the ACR Manual on MRI safety [38]. In addition, we note that the secondary magnetic field produced with the metasurface distorts the homogeneous field in the ROI created with an active RF shimming from the MR scanner. As a result, the MR image quality degrades, as seen in image quality assessment scores in Table S5 for Patient 8. The combined active and passive shimming performance might be addressed in further work. The passive shimming approach with a metasurface is promising as the structure can be used in any 3T MR system without an in‐built active shimming. Although we have covered a comprehensive range of BMIs both in numerical simulation as well as experimental studies, further studies are required to optimize the performance of the device in view of different BMI, gestational age, and posture of the subject.

Despite the indicated advantages, the study contains several limitations. The effect of the metasurface has not yet been evaluated in subjects with a low BMI during the first trimester of pregnancy, in case of multiple pregnancies, and in different subject positions, for example, left lateral posture. We plan to study these cases in the future; they might require a dedicated (tailored) metasurface. The optimization of the metasurface was very time‐consuming, limiting the design space (Metasurface Optimization in the Supporting Information), and it can be improved using neural networks [39]. Another strategy could include tunable elements in the structure to adjust the metasurface depending on the severity of the artifacts.

In this work, we have introduced and validated a passive shimming approach for improved fetal MRI at 3T using a flexible and compact metasurface. This sustainable approach significantly improves fetal imaging quality and diagnostic value compared to other passive and active shimming methods without compromising the subject's comfort level. The optimized structure successfully improves areas of reduced signal in the fetus, typically stemming from the standing wave effect. Moreover, numerical simulations with the proposed metasurface showed changes in SAR and temperature that did not exceed the required limits [30, 31]. Thus, the proposed approach is beneficial for improved diagnosis of fetal abnormalities at 3T MRI without requiring additional software and/or hardware changes.

## Supporting information


**Figure S1** Magnetic field distribution in three orthogonal central plains of the first resonant mode of the proposed metasurface. The field distribution was numerically calculated using an eigenmode solver.
**Figure S2** A, The numerical model for the preliminary calculation step includes the female body, fetus, fetal brain, and metasurface bent according to the body profile in the sagittal plane. B and C, B_1_^+^ field coefficient of variation (Cv) and mean value maps for optimization of the metasurface with fixed overall dimensions of 300 × 300 mm^2^ and changed capacitance and number of unit cells for the homogeneous female model at the 28^th^ gestational week. The region with optimal parameters is depicted with the white dotted lines. White star points to the best parameters of the metasurface.
**Table S1** Max SAR_10g_ in the fetal body, brain, placenta, and amniotic fluid. SAR results were normalized to the same B_1_^+^ for the reference case. All values are in W/kg units.
**Table S2** Results of numerically evaluated thermal distribution.
**Table S3** Numerically calculated results of B_1_^+^ field distribution for the fetal body and brain for 1 W of total accepted power.
**Figure S3** Comparison of the B_1_^+^ field distribution without and with an air gap between the body and pad.
**Figure S4** Numerically calculated B_1_^+^ field distribution for the voxel model at 28^th^ gestational weeks (7^th^ months of gestation) with the original metasurface and reduced one. The simulated field data are normalized to 1 W of accepted power. The reflection coefficient of the loop antenna was placed near the metasurfaces simulated in the CST, with capacitance in the range from 30 pF to 40 pF. The metasurface prototype was measured in the microwave lab. Exp ‐ experimental measurements, CST ‐ numerical simulations. Original MS ‐ metasurface with dimensions of 300 × 300 mm^2^, MS ‐ metasurface with dimensions of 280 × 280 mm^2^.
**Figure S5** T_2_‐weighted sagittal images for all patients without (first row) and with metasurface (second row).
**Figure S6** SNR images for patients at the 27^th^, 32^nd^, and 37^th^ gestation weeks (7^th^, 8^th^, and 9^th^ months).
**Table S4** Experimentally evaluated SNR in the fetal brain for all volunteers.
**Table S5** Image quality assessment (answers to questions A, B, and C) of the images without and with the metasurface using the Likert scale.

## Data Availability

The data that support the findings of this study are available from the corresponding author upon reasonable request.
