# Supplementary material for: Improved Fetal Magnetic Resonance Imaging Using a Flexible Metasurface
Source: NMR Biomed. 2025 Feb 25;38(4):e70016. doi: 10.1002/nbm.70016 (PMC11858842; doi:10.1002/nbm.70016)
Supplement: Supplementary file 1 — Figure S1 Magnetic field distribution in three orthogonal central plains of the first resonant mode of the proposed metasurface. The field distribution was numerically calculated using an eigenmode solver. Figure S2 A, The numerical model for the preliminary calculation step includes the female body, fetus, fetal brain, and metasurface bent according to the body profile in the sagittal plane. B and C, B1 + field coefficient of variation (Cv) and mean value maps for optimization of the metasurface with fixed overall dimensions of 300 × 300 mm2 and changed capacitance and number of unit cells for the homogeneous female model at the 28th gestational week. The region with optimal parameters is depicted with the white dotted lines. White star points to the best parameters of the metasurface. Table S1 Max SAR10g in the fetal body, brain, placenta, and amniotic fluid. SAR results were normalized to the same B1 + for the reference case. All values are in W/kg units. Table S2 Results of numerically evaluated thermal distribution. Table S3 Numerically calculated results of B1 + field distribution for the fetal body and brain for 1 W of total accepted power. Figure S3 Comparison of the B1 + field distribution without and with an air gap between the body and pad. Figure S4 Numerically calculated B1 + field distribution for the voxel model at 28th gestational weeks (7th months of gestation) with the original metasurface and reduced one. The simulated field data are normalized to 1 W of accepted power. The reflection coefficient of the loop antenna was placed near the metasurfaces simulated in the CST, with capacitance in the range from 30 pF to 40 pF. The metasurface prototype was measured in the microwave lab. Exp ‐ experimental measurements, CST ‐ numerical simulations. Original MS ‐ metasurface with dimensions of 300 × 300 mm2, MS ‐ metasurface with dimensions of 280 × 280 mm2. Figure S5 T2‐weighted sagittal images for all patients without (first row) and with metasurface (se [file NBM-38-e70016-s001.docx]

Supporting Information

**Improved Fetal Magnetic Resonance Imaging Using a Flexible Metasurface**

Vladislav Koloskov^1^, Viktor Puchnin^1^, Evgeniy Koreshin^1^, Anna Kalugina^1^, Wyger Brink^2^, Polina Kozlova^3^, Irina Mashchenko^3^, and Alena Shchelokova^1^*

1 School of Physics and Engineering, ITMO University, St. Petersburg 197101, Russian Federation

2 Magnetic Detection & Imaging Group, TechMed Centre, University of Twente, Enschede 7522 NH, The Netherlands

3 Department of Radiology, Federal Almazov North-West Medical Research Center, St. Petersburg 197341, Russian Federation

* Corresponding author at School of Physics and Engineering, ITMO University, St. Petersburg 197101, Russian Federation

e-mail: [a.schelokova@metalab.ifmo.ru](mailto:a.schelokova@metalab.ifmo.ru)

**Metasurface electromagnetic characteristics**

The proposed metasurface has the Fabry–Pérot resonances on a two-dimensional surface. Thus, several half-wavelengths are stacked along the orthogonal directions in the plane of the metasurface. In this work, a fundamental mode of the metasurface, i.e., half a wavelength (in *x* and *z* directions in Figure S1), is tuned to be above the operational frequency. This allows it to produce a homogenous secondary magnetic field at 123 MHz in the ROI. The magnetic field distribution at the first resonant frequency is shown in Figure S1.


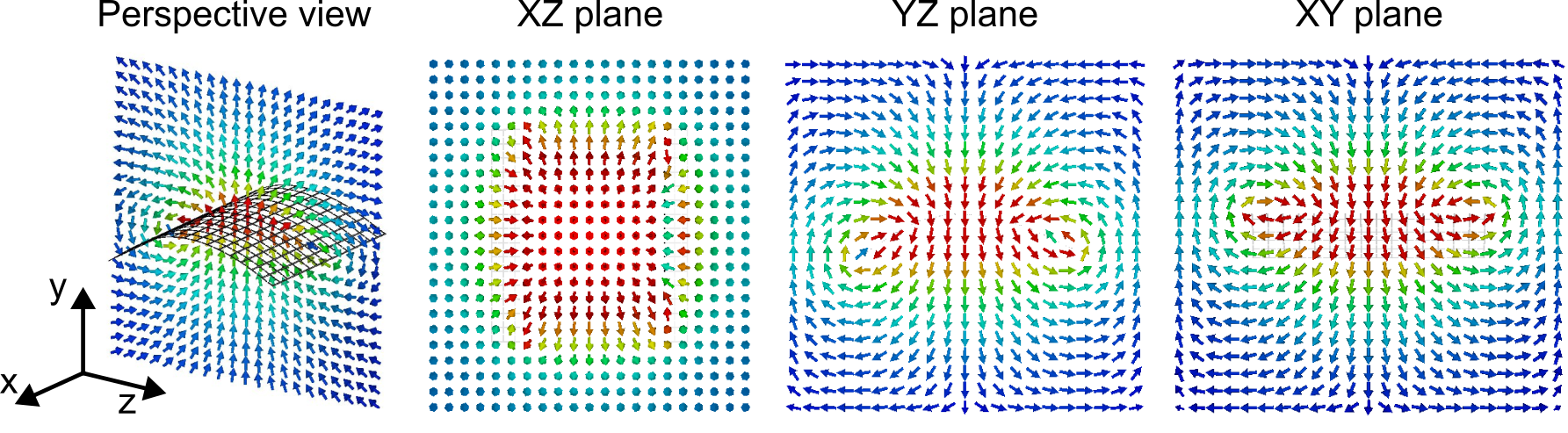


**Figure S1.** Magnetic field distribution in three orthogonal central plains of the first resonant mode of the proposed metasurface. The field distribution was numerically calculated using an eigenmode solver.

**Metasurface optimization**

The metasurface geometry is shown in Fig. S2A. The structure is a two-dimensional metal grid of cross-shaped cells connected via capacitors (parallel plate capacitors or lumped capacitors). During the metasurface geometry optimization, we aimed to maximize the B_1_^+^ field amplitude homogeneity (i.e., (1-Cv), where Cv - coefficient of B_1_^+^ field amplitude variation) in the fetal region. However, full-wave numerical calculation with voxels in CST Microwave Studio is time-consuming and requires significant computational resources, leading to difficulties in any optimization. Therefore, we simplified the female body model to overcome these difficulties and facilitate optimization. We replaced all tissues in the female body with a homogeneous material and converted it from voxel data to a bulk object. The same procedure was done for the fetal body and brain by replacing them with objects with corresponding averaged dielectric parameters. The main advantage of this simplified model is the ability to calculate it using a frequency domain (FD) solver for a single frequency instead of the time-consuming time domain (TD) calculation procedure for a frequency interval (the estimated required time is reduced by 1-2 orders in case the FD solver is used instead of TD solver, but voxel data is inappropriate in the first case).

The following algorithm was used to convert the voxel model of the body:

1. Select a voxel model layer number N.
2. Define a contour encapsulating all voxels of the female body tissue within that layer.
3. Extrude this contour to create a layer with a thickness equal to the thickness of the original layer N and the average dielectric parameter.
4. Repeat the procedure for voxel layer N+1.

The resulting model consists of layers with an external shape equal to the original voxel model, including the fetal body and brain. As a result, a complex voxel model is transformed into the ‘homogeneous model’ consisting of only three major homogeneous components (Fig. S2A). Averaged electromagnetic parameters have been indicated for each element of the homogeneous model according to ones from the corresponding voxel model.

The homogeneous model was placed inside a body birdcage-type coil. A generic RF transmit coil was modeled after a 16-leg high-pass whole-body birdcage coil with a 650 mm length and 700 mm diameter, with two quadrature excited feeds tuned and matched to 50 Ohms at 123 MHz. We added a curved layer of dielectric or metasurface pad to the model to study the influence of pads on B_1_^+^ field homogeneity. The solution to the electromagnetic problem occurs only at one point in the spectrum, namely at the Larmor frequency of 123 MHz. Before providing the optimization procedure, we ensured that changes in pad parameters minimally affected body coil performance, allowing one to neglect this effect and not perform coil matching or tuning procedures at each optimization step. We used only one voxel model at the 28^th^ gestational week (28GW), as it represents the averaged case with visible dielectric artifacts in the area of the fetus.


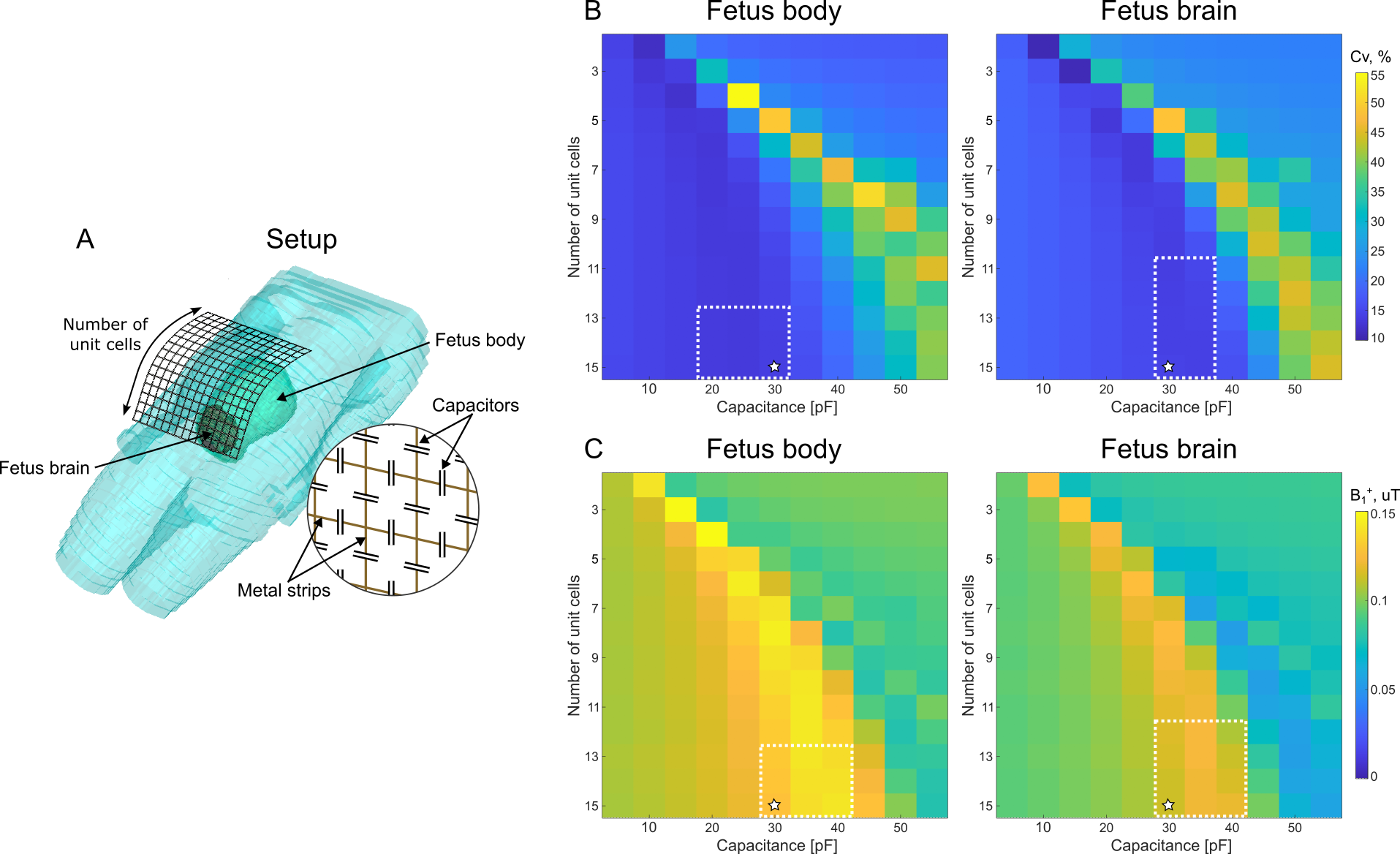


**Figure S2.** A, The numerical model for the preliminary calculation step includes the female body, fetus, fetal brain, and metasurface bent according to the body profile in the sagittal plane. B and C, B_1_^+^ field coefficient of variation (Cv) and mean value maps for optimization of the metasurface with fixed overall dimensions of 300×300 mm^2^ and changed capacitance and number of unit cells for the homogeneous female model at the 28^th^ gestational week. The region with optimal parameters is depicted with the white dotted lines. White star points to the best parameters of the metasurface.

Geometrical dimensions of the metasurface were selected, as described in the Methods section of the Main text, based on the reference dielectric pads used to improve fetal imaging in a particular area of interest of 292×299 mm^3^.^[1]^ We fixed the overall dimensions of the structure and changed the number of unit cells (N) from 2 to 15. Please note that the metasurface turns into a loop with N=1. We limited the maximum number of cells with 15×15 as further reducing the unit cell’s size may lead to additional electromagnetic coupling between patch-capacitors and copper strips (in PCB implementation) as they become comparable in size, as well as to a more rigid metasurface’s construction and loss in flexibility. We varied the capacitance for each setup from 5 pF to 55 pF using the 5 pF step.

The metasurface was bent to conform to the body profile and was positioned 5 mm away from it (Fig. S2A). The broadest transversal cross-section of the voxel model was selected to provide the bending procedure. Subsequently, the pad was bent according to the body curvature at a specific layer segment and was imported into CST using a macros approach. We investigated several positions of the pad on a human body. The pad’s location, either on the side of the body or the back, has minimal impact on field homogeneity in the fetal body and brain. The pad's location directly above the fetus (in the center of the abdomen) led to the greatest effect on the uniformity of the field. It is also possible to bend the pad in a sagittal plane, as shown in Fig. S2A, which was used for the following numerical simulation and optimization procedure.

The result of each calculation was the mean and inhomogeneity values of the B_1_^+^ field amplitude distribution, or Cv. Figure S2B,C shows Cv and mean B_1_^+^ value maps in the fetal body and brain when different metasurface configurations were used. The regions with the best Cv and mean B_1_^+^ values are shown with white dotted lines. The intersection of these areas is located at the points with C = 30 pF and N = 13-15, with the best case at N=15 unit cells.

**SAR results**

**Table S1.** Max SAR_10g_ in the fetal body, brain, placenta, and amniotic fluid. SAR results were normalized to the same B_1_^+^ for the reference case. All values are in W/kg units.

| Considered setup | Fetal body | Fetal brain | Placenta | Amniotic fluid |
| --- | --- | --- | --- | --- |
| **28^th^ gestational week** | | | | |
| Reference | 0.050 | 0.036 | 0.050 | 0.050 |
| Dielectric pad | 0.032 | 0.026 | 0.03 | 0.032 |
| Metasurface | 0.065 | 0.0437 | 0.065 | 0.065 |
| **36^th^ gestational week** | | | | |
| Reference | 0.050 | 0.050 | 0.050 | 0.050 |
| Dielectric pad | 0.036 | 0.036 | 0.036 | 0.036 |
| Metasurface | 0.063 | 0.063 | 0.063 | 0.063 |

**Thermal results with an air gap separation**

Additionally, we estimated the temperature more precisely by evaluating temperature distribution only in the fetal tissues and not in the rectangular FOV covering the fetus. In that case, the increase of max temperature is less than the recommended 2.4℃ [31] (Max-Max_ref_ column in Table S2), and the maximum possible increase (Max-Min_ref_ column in Table S2) is also below the recommendation. So, with an air gap between the dielectric pad and the subject, the increase in the temperature is less than in the case of metasurface.

**Table S2.** Results of numerically evaluated thermal distribution.

|  | Max, ℃ | Min, ℃ | Mean, ℃ | Std, ℃ | Max-Max_ref_, ℃ | Max-Min_ref_, ℃ |
| --- | --- | --- | --- | --- | --- | --- |
| **28^th^ gestational week** | | | | | | |
| Reference | 37.38 | 36.60 | 37.06 | 0.12 |  |  |
| Dielectric pad | 37.63 | 36.67 | 37.19 | 0.10 | 0.25 | 1.03 |
| Metasurface | 38.14 | 36.71 | 37.12 | 0.17 | 0.76 | 1.54 |
| **36^th^ gestational week** | | | | | | |
| Reference | 37.34 | 36.60 | 36.85 | 0.15 |  |  |
| Dielectric pad | 37.36 | 36.60 | 36.82 | 0.15 | 0.02 | 0.75 |
| Metasurface | 37.53 | 36.60 | 36.82 | 0.16 | 0.19 | 0.93 |

Also, we noticed that with a 5 mm air gap, the pad's performance has slightly worsened. In Table S3 and Figure S3 below, one can see that for 28GW with a spacer, the amplitude of the B_1_^+^ field in the fetal body and brain regions is lower compared to the case without the spacer. Based on the qualitative analysis of the images, even with the spacer, the distribution is better than in the reference case. However, for 36GW, the air gap significantly deteriorates the effect of the pad. Both qualitative and quantitative results show no difference between the case with a dielectric pad with a spacer and the reference case (without a dielectric pad).

**Table S3**. Numerically calculated results of B_1_^+^ field distribution for the fetal body and brain for 1 W of total accepted power.

|  | Fetal body | | Fetal brain | |
| --- | --- | --- | --- | --- |
|  | B_1_^+^, uT | Cv, % | B_1_^+^, uT | Cv, % |
| **28^th^ gestational week** | | | | |
| Reference | 0.133 | 20.6 | 0.147 | 17.9 |
| Dielectric pad without spacer | 0.183 | 22.5 | 0.230 | 8.2 |
| Dielectric pad with spacer | 0.168 | 20.4 | 0.197 | 11.7 |
| Metasurface | 0.170 | 18.5 | 0.174 | 15.6 |
| **36^th^ gestational week** | | | | |
| Reference | 0.102 | 19.4 | 0.111 | 9.6 |
| Dielectric pad without spacer | 0.125 | 25.9 | 0.118 | 5.8 |
| Dielectric pad with spacer | 0.100 | 26.9 | 0.094 | 8.3 |
| Metasurface | 0.104 | 31.5 | 0.095 | 8.3 |


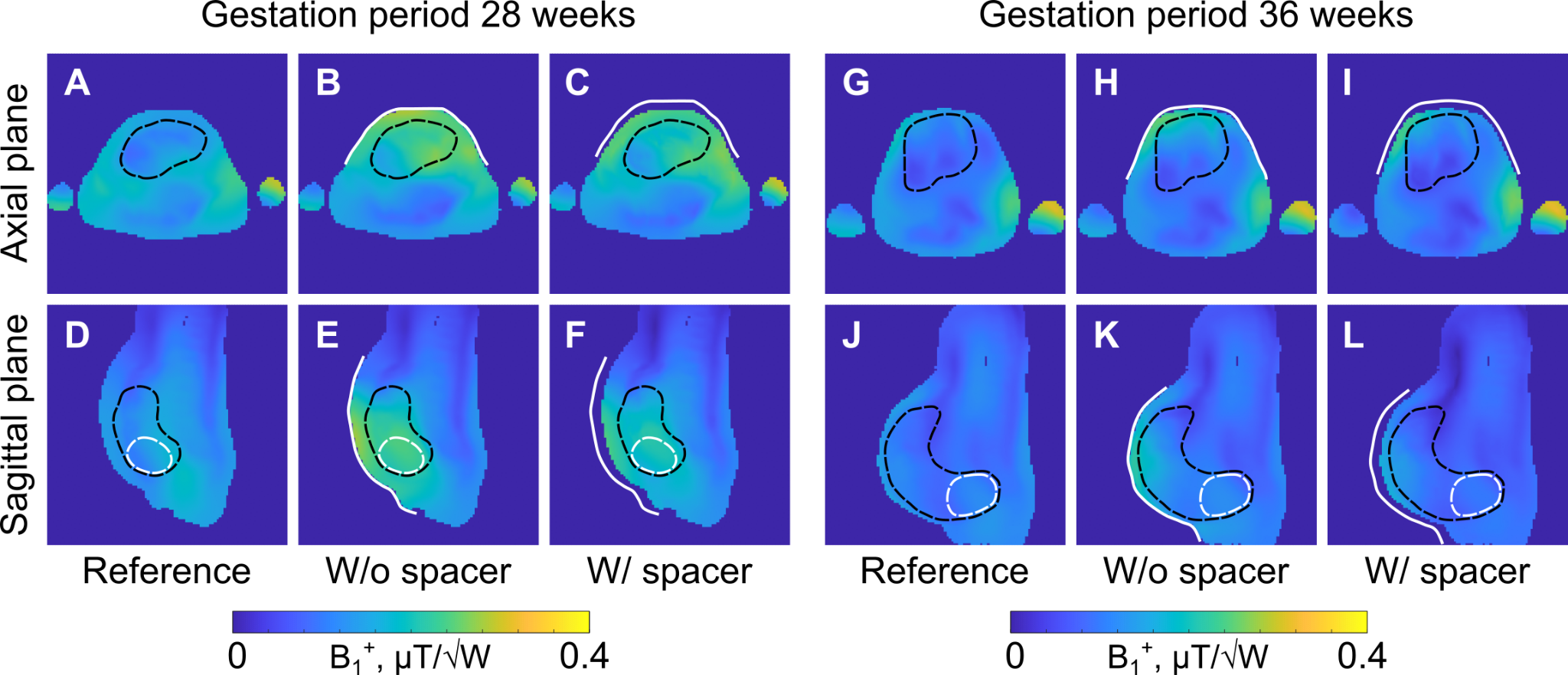


**Figure S3.** Comparison of the B_1_^+^ field distribution without and with an air gap between the body and pad.

**Metasurface prototype characterization**

During the optimization of the metasurface, the following parameters were selected as optimal for the highest B_1_^+^ field distribution homogeneity inside the fetal brain area: 15×15 unit cells, overall dimensions of 300×300 mm^2^, C = 30 pF. However, due to limitations imposed by the manufacturer of PCBs, the overall dimensions of the structure were reduced to 280×280 mm^2^. To achieve the same B_1_^+^ field redistribution in the ROI, we changed the number of unit cells from 15×15 to 12×12 with the same unit cell size of 20×20 mm^2^. We optimized the capacitance range from 30 to 40 pF to get the first resonance of the new structure at the same frequency as the original one (300×300 mm^2^). It was found that the setup with the capacitance of 37 pF was optimal.

We compared the effect of the metasurface with 15×15-unit cells (‘MS 30 pF’) and 12×12-unit cells (‘MS 37 pF’) on the B1+ field redistribution numerically. For B_1_^+^ estimation, the numerical setup was the same as before, with the whole-body birdcage coil used for field excitation. Both metasurfaces were centered on the fetus. Additionally, we estimated experimentally the reflection coefficient for a loop coil (with a diameter of 5 mm) placed 10 mm away from the unloaded metasurface. The loop coil was connected to the Vector Network Analyzer Obzor TR1300/1 (Copper Mountain Technologies, Indianapolis, IN).

Figure S4 shows almost identical B_1_^+^ field distributions in the sagittal and axial planes, with slightly higher B_1_^+^ values near the ‘MS 37 pF’. This effect is based on the fact that the first resonant frequency of the new metasurface is closer to the operating frequency of 123 MHz, is shown with numerically evaluated reflection coefficient for the old one structure (‘CST MS 30 pF’) and a new one (‘CST MS 37 pF’). So, one can see a 2 MHz shift when the structure was reduced in size. Nevertheless, numerical simulations showed that the first resonant frequency for ‘MS 30 pF’ and ‘MS 37 pF’ is sufficiently separated from the ^1^H Larmor frequency at 3 T (123 MHz).

Once the new metasurface was manufactured, we additionally measured the reflection coefficient and compared it with the results from the simulation. One can see in Figure S4 that a 7 MHz shift was found for the first resonant frequency of the manufactured metasurface (‘Prototype 37 pF’) compared to the one from simulation (‘CST MS 37 pF’) due to some small deviations in the relative permittivity value of the substrate used in fabrication.


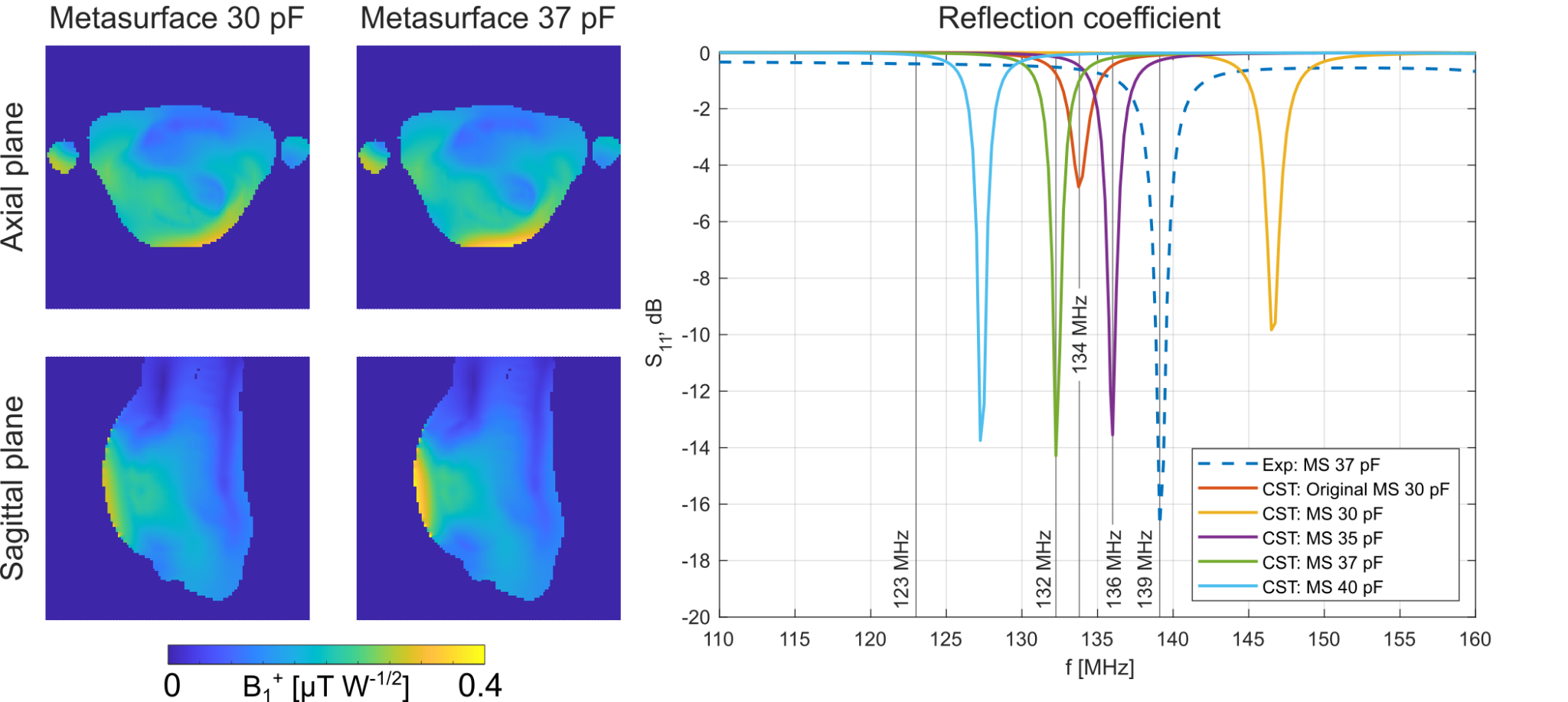


**Figure S4.** Numerically calculated B_1_^+^ field distribution for the voxel model at 28^th^ gestational weeks (7^th^ months of gestation) with the original metasurface and reduced one. The simulated field data are normalized to 1 W of accepted power. The reflection coefficient of the loop antenna was placed near the metasurfaces simulated in the CST, with capacitance in the range from 30 pF to 40 pF. The metasurface prototype was measured in the microwave lab. Exp - experimental measurements, CST - numerical simulations. Original MS - metasurface with dimensions of 300×300 mm^2^, MS - metasurface with dimensions of 280×280 mm^2^.

**Image Analysis**

Figure S5 shows T_2_-weighted sagittal images for all patients without and with metasurface. In all cases one can see the improvement of image quality in the region of the fetal brain.

Figure S6 shows SNR maps plotted by dividing T_2_-weighted anatomical images (Fig. 5 from the main text) by the standard deviation of noise measured in the image corners (signal-free areas) and then multiplied by the 0.66 Rayleigh distribution correction factor. One can significantly improve sagittal and transverse planes for all shown patients.

Additionally, we estimated SNR in the fetal brain region for the whole study population for quantitative analysis of SNR results. These values shown in Table S4 were calculated as the mean SNR values in the region of the fetal brain in sagittal planes. For all patients, the change in SNR was positive from 10.0% to 49.5%. The qualitative image analysis is provided in Table S5.


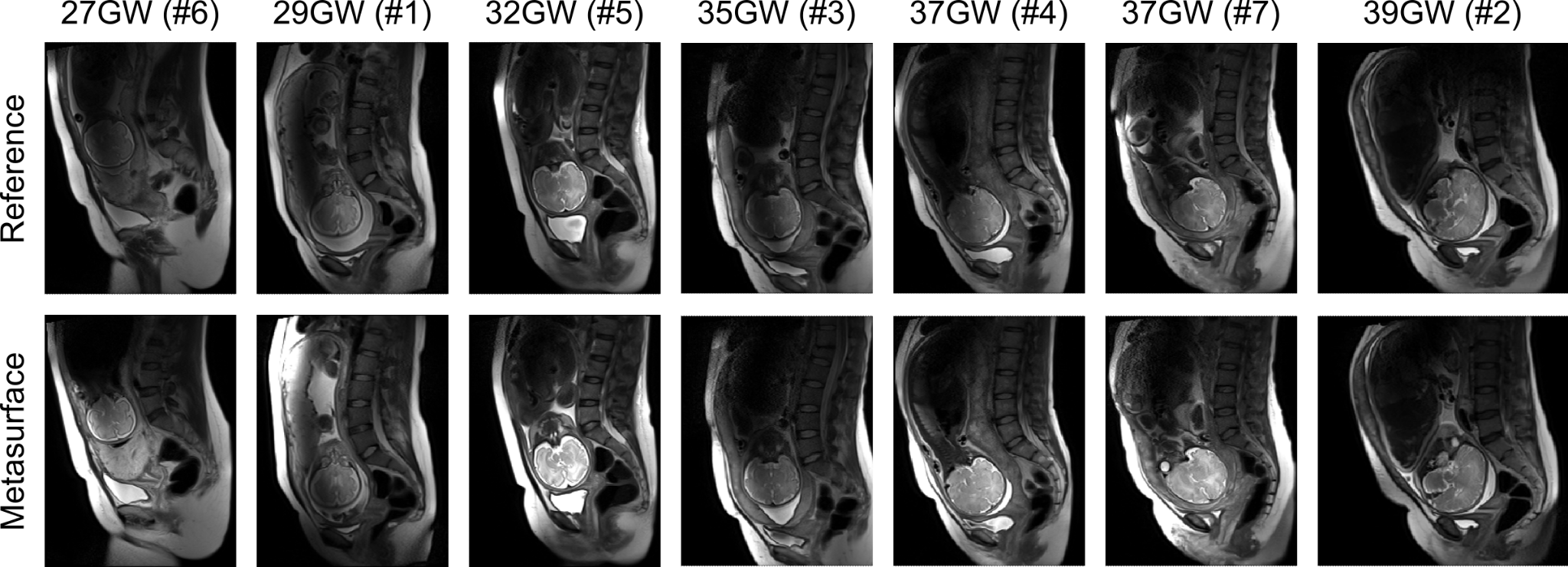


**Figure S5.** T_2_-weighted sagittal images for all patients without (first row) and with metasurface (second row).


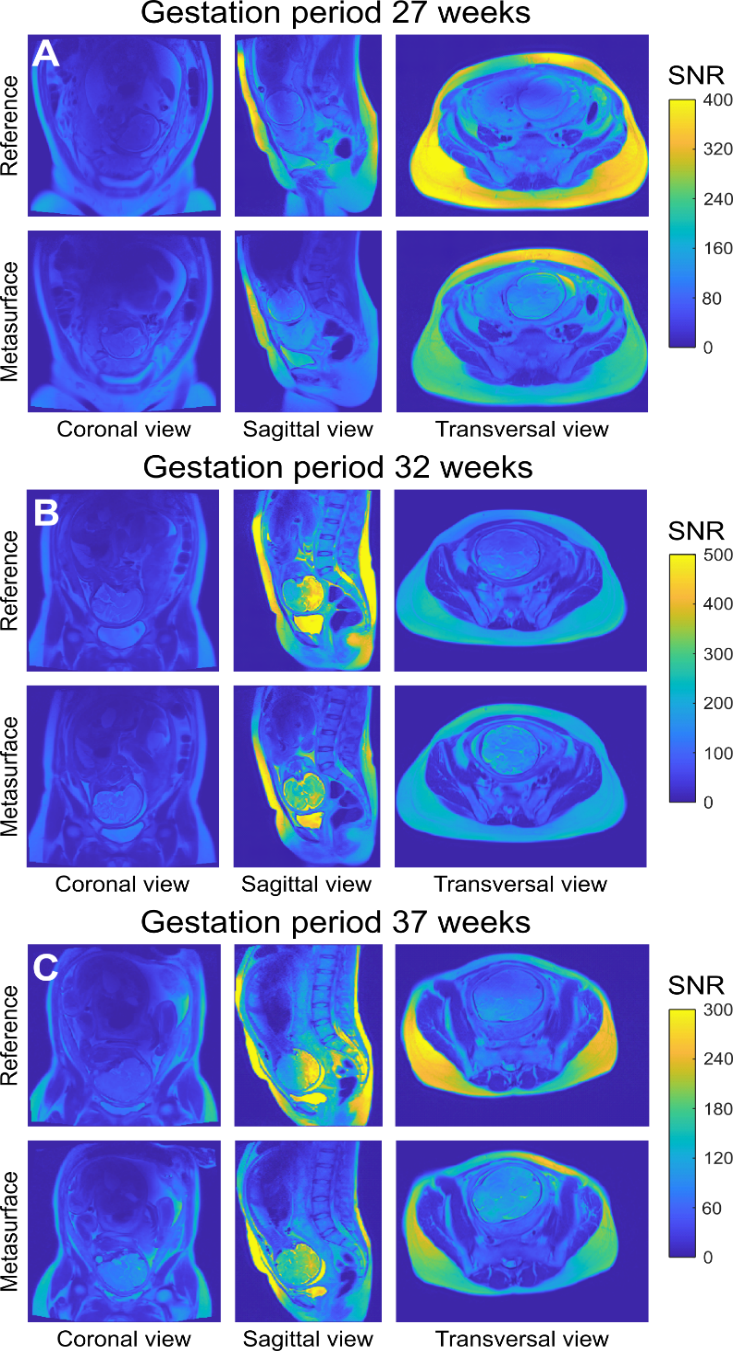


**Figure S6.** SNR images for patients at the 27^th^, 32^nd^, and 37^th^ gestation weeks (7^th^, 8^th^, and 9^th^ months).

**Table S4.** Experimentally evaluated SNR in the fetal brain for all volunteers.

| Patient ID | Gestational week | SNR, fetal brain | |
| --- | --- | --- | --- |
|  |  | Without metasurface | With metasurface |
| 1 | 29 | 391 | 499 |
| 2 | 39 | 355 | 426 |
| 3 | 35 | 156 | 206 |
| 4 | 37 | 209 | 230 |
| 5 | 32 | 477 | 587 |
| 6 | 27 | 196 | 293 |
| 7 | 37 | 285 | 347 |
| 8 | 32 | 456 | NA |

**Table S5.** Image quality assessment (answers to questions A, B, and C) of the images without and with the metasurface using the Likert scale.

| Expert No. | Patient ID | 6-channel body coil | | | 6-channel body coil & metasurface | | |
| --- | --- | --- | --- | --- | --- | --- | --- |
|  |  | A | B | C | A | B | C |
| Expert 1 | 1 | 4 | 3 | 4 | 4 | 5 | 5 |
|  | 2 | 3 | 3 | 3 | 5 | 4 | 5 |
|  | 3 | 4 | 4 | 3 | 5 | 5 | 5 |
|  | 4 | 4 | 4 | 4 | 4 | 4 | 5 |
|  | 5 | 4 | 4 | 3 | 5 | 5 | 4 |
|  | 6 | 3 | 4 | 4 | 5 | 5 | 5 |
|  | 7 | 2 | 3 | 4 | 4 | 5 | 5 |
|  | 8 | 5 | 4 | 5 | 4 | 4 | 5 |
|  | Mean | 3.63 | 3.63 | 3.75 | 4.50 | 4.63 | 4.88 |
|  | Std | 0.86 | 0.48 | 0.66 | 0.50 | 0.48 | 0.33 |
|  |  |  |  |  |  |  |  |
| Expert 2 | 1 | 3 | 4 | 4 | 4 | 4 | 5 |
|  | 2 | 2 | 3 | 4 | 5 | 4 | 4 |
|  | 3 | 4 | 4 | 4 | 5 | 4 | 4 |
|  | 4 | 3 | 4 | 3 | 5 | 4 | 4 |
|  | 5 | 4 | 3 | 4 | 4 | 4 | 4 |
|  | 6 | 4 | 3 | 3 | 4 | 5 | 5 |
|  | 7 | 2 | 3 | 4 | 4 | 5 | 5 |
|  | 8 | 4 | 5 | 5 | 4 | 4 | 4 |
|  | Mean | 3.25 | 3.63 | 3.88 | 4.38 | 4.25 | 4.38 |
|  | Std | 0.83 | 0.70 | 0.60 | 0.48 | 0.43 | 0.48 |
|  |  |  |  |  |  |  |  |
| Expert 3 | 1 | 4 | 4 | 4 | 5 | 5 | 5 |
|  | 2 | 3 | 3 | 4 | 4 | 4 | 5 |
|  | 3 | 4 | 4 | 3 | 5 | 5 | 4 |
|  | 4 | 3 | 4 | 4 | 4 | 5 | 5 |
|  | 5 | 4 | 4 | 4 | 5 | 5 | 4 |
|  | 6 | 3 | 4 | 3 | 4 | 5 | 5 |
|  | 7 | 2 | 4 | 4 | 4 | 4 | 4 |
|  | 8 | 5 | 5 | 5 | 3 | 4 | 4 |
|  | Mean | 3.50 | 4.00 | 3.88 | 4.25 | 4.63 | 4.50 |
|  | Std | 0.87 | 0.50 | 0.60 | 0.66 | 0.48 | 0.50 |

Letters A, B, and C mark the corresponding questions to evaluate images:

1. Please evaluate the general diagnostic quality of the images - can these images be used in clinical practice?
2. Please evaluate the quality of the images in terms of the absence/presence of a dielectric artifact.
3. Please evaluate the images regarding the visualization quality of the extrafetal and fetal structures.

**References**

1. van Gemert J, Brink W, Remis R, Webb A. A simulation study on the effect of optimized high permittivity materials on fetal imaging at 3T. *Magnetic resonance in medicine*. 2019;82(5):1822-1831.
